# Blended teaching versus traditional teaching for undergraduate physiotherapy students at the University of the Witwatersrand

**DOI:** 10.4102/sajp.v77i1.1544

**Published:** 2021-05-17

**Authors:** Sadiya Ravat, Paula Barnard-Ashton, Monique M. Keller

**Affiliations:** 1 Department of Physiotherapy, School of Therapeutic Sciences, Faculty of Health Sciences, University of the Witwatersrand, Johannesburg, South Africa; 2 eFundanathi, School of Therapeutic Sciences, Faculty of Health Sciences, University of the Witwatersrand, Johannesburg, South Africa

**Keywords:** blended learning, higher education, feasibility, teaching, COVID-19, physiotherapy, undergraduate learning

## Abstract

**Background:**

Shifting from face-to-face teaching to incorporating technology may prepare students better for future work as health professionals. Evidence of blended teaching’s effect on the academic performance of undergraduate physiotherapy students’ performance is scarce.

**Objective:**

The purpose of our study was to determine students’ theoretical and clinical performance in a blended teaching module compared to their own performance in two knowledge areas taught face to face, and student perceptions of blended teaching in the third-year physiotherapy curriculum.

**Methods:**

The cross-sectional study design included 47 third-year physiotherapy students. The orthopaedic module was delivered using a blended teaching approach in two consecutive semesters, whilst two other physiotherapy knowledge areas, neuromusculoskeletal and cardiopulmonary, in the same semesters were delivered face to face. Theoretical and clinical performances of students were compared for significance and effect. Students were assessed on their theoretical and clinical knowledge in all areas using the same assessment methods. The students (*n* = 43) also completed a survey on their blended teaching experience.

**Results:**

Significantly higher theoretical marks for orthopaedics were calculated compared to neuromusculoskeletal and cardiopulmonary for both semesters with a large positive effect (average Cohen *d* = 4.44) for blended teaching on theoretical examination performance; no statistically significant difference for clinical performances. Students felt engaged in the blended teaching process, and 72% preferred blended teaching over face-to-face teaching or online delivery.

**Conclusion:**

Blended teaching improved the theoretical marks, demonstrating that knowledge acquisition was improved, but not clinical performance.

**Clinical implications:**

The study contributes to the knowledge base of blended learning in Health Science Education in South Africa. The authors identified a gap where future studies should investigate the effect of blended learning on clinical performance outcomes as a continuation from this one.

## Introduction

The coronavirus disease 2019 (COVID-19) pandemic caused an instant shift in teaching across the world. A shift from traditional face-to-face teaching to e-learning or a blended teaching approach occurred. Even prior to COVID-19, blended learning has rapidly grown in education (Vallée et al. 2020).

To adequately prepare students for a changing workforce, educators need to reflect on their teaching strategies to incorporate the 21st-century learning skills of critical thinking, collaboration, communication, innovation and creativity, independent and self-directed learning, and using technology to learn (Kennedy & Heineke 2014; Kereluik et al. 2013; Little 2013). Traditional face-to-face teaching approaches have been, and are still being, used in health sciences education to prepare undergraduate students with the necessary theoretical knowledge as well as clinical skills to enter a clinical work environment. However, technology has changed the teaching and learning culture (Department of Education 2004; Hämäläinen, Kiili & Smith 2017). Since the emergence of technology-based teaching platforms, electronic learning (e-learning) has increased in popularity, and traditional teaching approaches have been augmented with an e-learning component (Department of Education 2004; Liu et al. 2016). Higher educational institutions are increasingly incorporating e-learning into health education in a blended teaching format (Means et al. 2013), and authors have even called the blended mode of teaching the ‘new normal’ (Norberg, Dziuban & Moskal 2011).

Blended teaching incorporates the traditional face-to-face lecturing style with a synchronous or asynchronous e-learning component (Garrison & Kanuka 2004; Liu et al. 2016). It is distinctly different from online teaching, where there is no face-to-face component. The strength of a blended teaching approach lies in the collective advantages of both face-to-face and e-learning approaches (Wu, Tennyson & Hsia 2010). In traditional face-to-face lectures, although there is the cost of transport and the time of each student participating in the lecture, a sense of community is fostered (Kemp & Grieve 2014). E-learning has the advantages of saving transport costs, the convenience of learning remotely and up-to-date information being available at the touch of a button (Liu et al. 2016). The strengths of blended learning, underpinned by Siemens’ 2006 theory of connectivism, lie in the creation of an extended community, whereby students can engage in dialogue, debate and have open lines of communication with experts and the global community (McDonald et al. 2014; Siemens 2006). The extended community can be built into blended teaching by means of reflection, discussion groups, debates and seeking of information in small groups. This fosters critical thinking and reflection; supports flexibility, independence and collaborative learning; and enhances positive motivation amongst students (McDonald et al. 2014; López-Pérez, Pérez-López & Rodríguez-Ariza 2011).

Liu et al. (2016) conducted a systematic review on the effectiveness of a blended teaching approach compared to no intervention, traditional face-to-face and e-learning approaches. Although high article heterogeneity and publication bias were concerns, the pooled effect size of 0.81 indicated that a blended learning approach may be more effective than traditional lecture-based or e-learning only for acquiring knowledge amongst healthcare students (Liu et al. 2016). Stander, Grimmer and Brink (2019) conducted a systematic scoping review exploring the learning styles amongst physiotherapy undergraduate students (*n* = 910), postgraduate students (*n* = 361) and qualified physiotherapists (*n* = 23) over a 26-year period. Students included in the time period of the review sourced information from traditional sources which has since shifted to learning electronically. There was inconsistent evidence on how learning amongst physiotherapy students occurs, especially in developing countries. In conclusion, Stander, Grimmer and Brink stated that active learning with a clear understanding of theoretical concepts through a blended learning approach may guide physiotherapy students’ learning.

Knowledge acquisition is a crucial part of students’ learning in health education. Vallée et al. (2020) evaluated the effectiveness of blended teaching on knowledge acquisition in a systematic review and meta-analysis. Albeit a wide variety of different blended teaching variants were included, consistent improved knowledge outcomes were seen for students receiving blended teaching compared to traditional teaching. A recommendation of further studies to confirm the improved knowledge outcomes for blended teaching is answered with our study. Students in the third year of their undergraduate physiotherapy training at our university enter the clinical field of orthopaedics for the first time. This may be a challenging experience whereby they must integrate theoretical knowledge into practical skills, and blended teaching could ease this transition. This premise is supported by Motsumi, Bedada and Ayane (2019), who found that blending their traditional lecture-based surgical skills training with Moodle, which hosted 3D aminations, resulted in significantly higher pre-post-test knowledge impact scores and high learning satisfaction compared to the traditionally taught group. Barnard-Ashton, Koch and Rothberg (2014) investigated the influence of blended teaching on student performance in the undergraduate occupational therapy curriculum at the University of the Witwatersrand. They showed that when students had a significantly higher access footprint to the e-learning content of their course, there was a small but relevant positive effect (average *d* = 0.31) on student performance (Barnard-Ashton et al. 2014). In their systematic literature review aimed at determining the role of blended teaching approaches on healthcare students’ clinical education, Rowe, Frantz and Bozalek (2012) found that the gap between theoretical knowledge and clinical practice can be bridged through blended teaching. However they concluded that there is a need for future research to establish the use of a blended teaching approach and how it impacts students’ clinical practice, further supporting the need for our study.

Our study aimed to determine the effect of blended teaching compared to traditional face-to-face teaching approaches and gauge the perceptions of students regarding learning through a blended teaching approach. What makes our study more pertinent is the change that has occurred since the start of the COVID-19 pandemic. Lecturers face the challenge of reimagining their teaching, where blended teaching may become the ‘new normal’. Understanding the impact and feasibility of a blended teaching approach may be useful for lecturers to inform future teaching styles, as well as enhance learning for their students.

## Methods

A cross-sectional study included a convenience sample of third-year undergraduate physiotherapy students at the University of the Witwatersrand. Physiotherapy students in their third year are divided into two groups for the year and change over the knowledge areas taught between the first and second semester in order to manage the class size and clinical placement burden. One group (half of the class) (*n* = 24) was taught orthopaedic physiotherapy in January and February, and the other (*n* = 23) was taught orthopaedic physiotherapy in July. Both groups were taught the same orthopaedics content using a blended teaching approach. Third-year physiotherapy students who were repeating their year of study were excluded.

The blended learning programme consisted of a revision quiz, online activities and face-to-face lectures. The online activities consisted of videos sourced from the internet, podcasts, group case studies and online quizzes in lesson plans on Moodle, the learning management system that is used in the School of Therapeutic Sciences at the University of the Witwatersrand. The content was constructed by the orthopaedics lecturer with the help of a blended learning expert. The concepts were broken down into components and the online content was constructed based on the level of understanding required for each concept. In addition to the face-to-face lectures, there was a face-to-face debate task. In total there were six online components, eight face-to-face lectures and the face-to-face debate task. The face-to-face teaching covered four general orthopaedic lectures (complications of fractures, principles of fracture management, orthopaedic radiology and amputations), one lower limb lecture (distal femur, tibia, fibula and ankle fractures), two upper limb lectures (pathologies, fractures and dislocations of the shoulder, distal forearm, wrist and hand, and hand injuries) and an arthroplasty lecture. At the end of the blended teaching orthopaedic teaching period, the students were taken to one of the academic hospitals, where they were orientated to the clinical setting, and they had the opportunity to assess and treat patients under supervision. They were divided into groups of two and tasked to compile a video of management of their patients (with permission), which was to be shown to the rest of the class.

For the neuromusculoskeletal (NMS) and cardiopulmonary (CP) courses, physiotherapy knowledge areas covered in the same semester as the orthopaedic area, only traditional face-to-face lectures were used. The CP knowledge area consisted of 13 face-to-face lectures and three practical sessions. The NMS knowledge area consisted of two lectures and two practical sessions. At the end of the teaching period in both CP and NMS, and similar to orthopaedics, the students were taken to a clinical placement area, where they were orientated to the clinical area and assessed and treated patients under supervision.

The performance of the students on all knowledge areas was evaluated through a knowledge test at the end of the teaching block, and a clinical performance mark was given at the end of the clinical placement.

### Study instrument and procedure

After giving informed consent, students completed a questionnaire based on the work of Owston, York and Murtha (2013) that was developed using REDcap (Research Electronic Data Capture), which is a secure, web-based software platform designed to support data capture for research (Harris et al. 2009, 2019). This questionnaire was used in a study by Owston et al. (2013), which similarly assessed the perceptions and performance of students doing blended learning in a university environment. The questionnaire was compiled from other blended learning questionnaires. It scored a high reliability of 0.908 (Cronbach’s alpha coefficient). The questionnaire assessed the student perspectives regarding blended teaching compared to a traditional teaching approach during the preclinical teaching block. A five-point Likert scale allowed students to select between 1 (strongly agree), 2 (agree), 3 (neutral), 4 (disagree) and 5 (strongly disagree). The blended teaching section covered aspects of engagement with content, interaction, understanding, access to resources, reflection opportunities, usage of technology and course factors. The questionnaire ended with questions investigating which teaching style was favoured by the students.

The results of one theoretical examination and a summative clinical mark were obtained for undergraduate students (*n* = 47) in the field of orthopaedics. These results were entered onto an Excel spreadsheet, and statistical analysis was performed. The marks for the NMS and CP physiotherapy knowledge areas that were taught by traditional face-to-face lectures within the same semester were recorded and acted as paired comparison data (of the students’ own marks) to their orthopaedic performance mark.

### Statistical analysis

Descriptive statistics were undertaken to reduce the data, and a two-tailed Student’s *t*-test was used to determine if there was a significant difference (α = 0.05) between the orthopaedic marks and the marks obtained in the NMS and CP areas for both the theoretical examination and the summative clinical assessment. Cohen’s *d* was applied to determine direction of difference and effect size, where 0.2 is considered a small effect, 0.5 a medium effect and > 0.8 a large effect (Ellis 2009). Likert scale responses to the survey were descriptively analysed.

### Ethical considerations

This study was approved by the Medical Human Research Ethics Committee of the University of the Witwatersrand, the Dean of Student Affairs and the Head of the Department of Physiotherapy (ethical clearance number M170571). All participants gave informed consent prior to taking part in our study.

## Results

### Theoretical and clinical performance

The theoretical examination marks of the two groups of students in the orthopaedics area were similar between the two semesters (Table 1). When comparing the performance of students in orthopaedics in both semesters, when the blended teaching approach was used, to their own performance in NMS and CP areas, the students performed significantly higher in the theoretical examination on the orthopaedics content. This is further evidenced by the large positive effect of blended teaching on the theoretical examination performance over their own performance on theoretical examinations on content delivered by conventional teaching methods (average Cohen’s *d* = 4.44).

**TABLE 1:** Theoretical examination results: Mean standard deviation and effect size.

Variable	First semester (*n* = 24)			Second semester (*n* = 23)		
	Orthopaedic (blended)	NMS (conventional)	Cardiopulmonary (conventional)	Orthopaedic (blended)	NMS (conventional)	Cardiopulmonary (conventional)
Mean	79.7	65.7	65.2	80.1	57.0	44.7
Standard deviation	4.4	5.7	4.7	4.0	5.4	6.0
*p*	-	0.001*	0.000*	-	0.000*	0.000*
Cohen’s *d*	-	2.75	3.19	-	4.86	6.94
Effect *r*	-	0.81	0.85	-	0.92	0.96

* , significant result.

NMS, neuromuculoskeletal.

In the clinical summative assessment marks (Table 2), there was no statistically significant difference between students’ orthopaedics clinical performance assessment in both semesters when compared with their performance in the NMS and CP areas. An average small negative effect (average Cohen’s *d* = −0.26) was evident, indicating that the students performed slightly worse on their clinical orthopaedics assessments than on their other two specialities.

**TABLE 2:** Clinical summative results: Mean and standard deviation.

Variable	First semester (*n* = 24)			Second semester (*n* = 23)		
	Orthopaedic (blended)	NMS (conventional)	Cardiopulmonary (conventional)	Orthopaedic (blended)	NMS (conventional)	Cardiopulmonary (conventional)
Mean	73.5	77.1	79.8	73.1	72.3	75.1
Standard deviation	12.6	8.9	9.0	8.7	6.8	9.6
*p*	-	0.29	0.07	-	0.26	0.47
Cohen’s *d*	-	−0.33	−0.58	-	0.10	−0.22
Effect *r*	-	−0.16	−0.28	-	0.05	−0.11

NMS, neuromuculoskeletal.

### Student perceptions of blended teaching

In the blended teaching questionnaires, the Likert scale ranged from 1 (strongly agree) to 5 (strongly disagree). Of the students who consented to participate in our study, 43 (91.5%) completed the survey on their perception of blended teaching. The survey covered three aspects: engagement and affect, knowledge and learning, and the blended teaching process.

**FIGURE 1:**
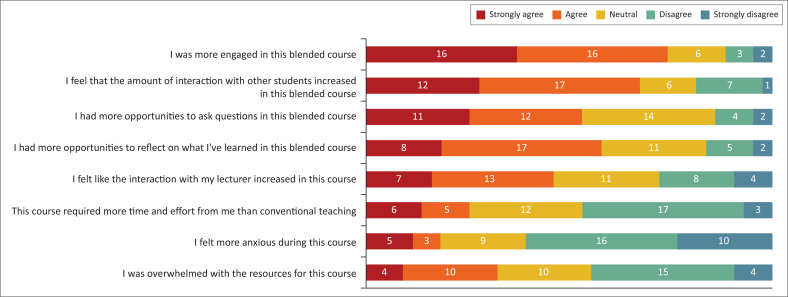
Engagement and effect of the students during the blended course.

Thirty-two students (74.4%) felt that they were more engaged; 67.4% felt that the amount of interaction with other students increased, whilst 46.5% felt that interaction with the lecturer increased through the blended teaching approach (Figure 1). Whilst 32.6% of students felt overwhelmed by the resources in the course, only 16.3% felt that blended teaching made them more anxious.

**FIGURE 2:**
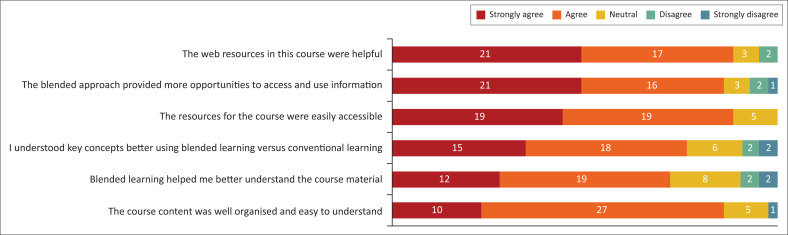
Student perceptions of knowledge and teaching.

Questions relating to knowledge and learning can be seen in Figure 2. The students predominantly agreed or strongly agreed that the web resources were helpful, that a blended approach to teaching provided more opportunities to use and access information, that resources were easy to access, that they understood concepts better and that their understanding of the course material was improved. Only one student disagreed (strongly) that the blended course content was well organised and easy to understand.

**FIGURE 3:**
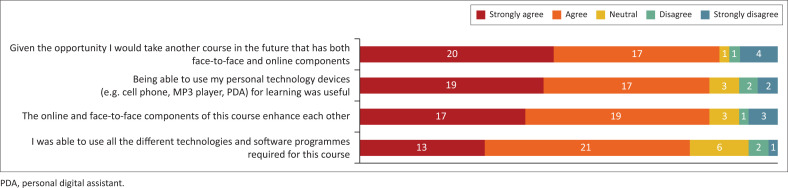
Student perceptions of the blended teaching process.

The majority of students indicated that using their devices for learning was useful (83.7%) and that they were able to use the technology and software needed to complete the course (79.1%). The students (83.7%) also believed that the online and face-to-face components of the course enhanced each other, and 86% agreed that they would take another blended teaching course if given the opportunity (Figure 3).

**FIGURE 4:**
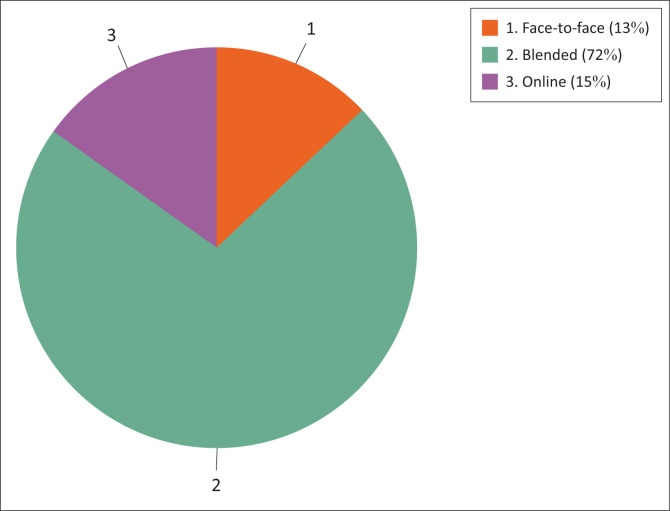
Student preferences after doing the blended teaching module.

Seventy-two per cent of students indicated that they would prefer a blended teaching approach as indicated in Figure 4.

When asked to explain their preference of teaching style, one student responded, ‘I enjoy being able to learn on my own but still have the opportunity to have things explained or confirmed by the lecturer and the opportunity to ask questions’ (Participant number [PN] 3, female, physiotherapy student). Further students voiced their perspectives: ‘I feel that not every lecture has to be given face to face, but some things require explanation in person’ (PN 11, male, physiotherapy student), ‘More convenient in terms of not having to sit in 1.5 hours of traffic daily! Also, more importantly I am able to grasp concepts better watching podcasts as I can rewind, whereas in a lecture it is difficult to always ask the lecturer to repeat herself’ (PN 18, male, physiotherapy student). Students preferring face-to-face teaching voiced their opinions; ‘easier to ask questions and discuss the content’ (PN 14, female, physiotherapy student) face-to-face, ‘online does not emphasise interaction, participation, attention, it does not allow us to ask ‘ (PN 24, female, physiotherapy student).

### Perceptions on discussion sessions

The students were asked whether they preferred a classroom or an online discussion. Six students preferred classroom discussions, stating that they had ‘more interaction and [found it] easier to focus and learn’ (PN 14, female, physiotherapy student). Eight students preferred online discussions, stating that ‘podcasts are amazing; it was really helpful, and I can always go back when revising and when studying lecture material; I can study while listening’ (PN 25, male, physiotherapy student). The remaining respondents preferring a combination of online and classroom discussion, stating that ‘both have great benefits and enhance my learning’ (PN 28, female, physiotherapy student).

### Perceptions of blended teaching and the least useful aspects

The students were asked which aspects of the blended course they found least useful. At times the students experienced poor Wi-Fi and internet connection, posing challenges, made apparent by a student responding ‘being dependent solely on technology, when the server crashed, we were unable to learn’ (PN 13, male, physiotherapy student). A preference for podcasts over YouTube lectures was mentioned. One student preferred ‘the YouTube videos that were not done by the lecturer’ (PN 22, female, physiotherapy student) and noted that ‘it took a lot of time at home; it was easier to look at podcasts than the YouTube videos that were online’ (PN 21, female, physiotherapy student).

### Perceptions of blended teaching and the most useful aspects

When the students were asked which aspect of the blended course they found most useful, they answered, ‘I only enjoyed working on my own as I was able to start past paper questions in the same time rather than sitting in a classroom’ (PN 45, female, physiotherapy student).

‘[*T*]he ability to go through the work at a pace that suited me, as well as having the ability to ask the lecturer a question when having a lecture face-to-face.’ (PN 43, female, physiotherapy student)

‘Hospital visit and practical on amputation’ (PN 30, female, physiotherapy student), ‘online and group discussions’ (PN 25, male, physiotherapy student) and ‘podcasts’ (PN 26, female, physiotherapy student). The students mentioned that they preferred the lecturer podcasts to YouTube videos.

## Discussion

Our study compared a blended teaching approach in an orthopaedics module to two physiotherapy modules taught face to face. All three modules were offered in the same semester, over two consecutive semesters.

The students scored significantly higher theoretical marks in both semesters for the orthopaedics module, showing an average large effect (average *d* = 4.44) of blended teaching over the face-to face approach. This is similar to the findings of a systematic review conducted by Liu et al. (2016), where blended teaching approaches were found to be more effective or as effective when compared to a face-to-face approach or purely e-learning teaching. Vallée et al. (2020) study supports the finding that blended teaching and learning have consistently superior effects on health education outcomes with different blended design variants. When comparing the summative clinical marks between the three different physiotherapy knowledge areas, however, there was no significant difference and little effect, indicating that the transfer of knowledge to the clinical setting was not improved by the higher theoretical marks achieved in the blended teaching module. Whilst these results contribute to our understanding of the impact of blended teaching on clinical performance, as suggested by Rowe et al. (2012), we need further research on the factors that contribute to this outcome.

Regarding the students’ perceptions of blended teaching, predominantly positive responses were seen for engagement, interaction and helpful resources, and the students indicated that the blended teaching provided them more opportunity to access and use information. The students also felt that their understanding of key concepts was improved with the blended teaching approach, and this is evident with the higher theoretical examination marks obtained for orthopaedics. They agreed that the blended teaching process was clear and organised to support their learning. Not having a pure online teaching approach but including face-to-face lectures was in retrospect a good decision, as 72% of students indicated that they preferred the blended teaching module. Consideration should, however, be given to the 14 students (32.6%) who indicated that they were overwhelmed with the resources; a possible reason for this may be that it was the first time orthopaedic or physiotherapy content was delivered in a blended format. Connectivity and Wi-Fi issues interrupted the students’ teaching, but the students agreed that what made blended learning useful was the fact that the material could be revisited at any time, making connectivity a problem that can be overcome with lesson plans and podcasts. In the blended learning orthopaedic teaching module, the same benefits of e-learning, namely convenience and transcending the boundaries of space and time (Liu et al. 2016:e2), provided students with the latest information at their fingertips, and the same 21st-century skills of collaborative interaction were observed by the authors as what was found in other studies (Wu et al. 2010:155–164; Peng et al. 2014:16).

Other 21st-century skills included the teacher being a facilitator and students having to do self-directed learning, whereby they were responsible for doing the online activities themselves. At the end of the activities, there were quizzes to test their knowledge of the lesson. This was a huge change for the students as they were accustomed to just presenting themselves for a lecture. Self-directed learning is an important skill for students, as when they are qualified health professionals they will be responsible for their own continuous professional development. The group case discussions also fostered collaboration and communication, with the critical thinking and creativity of finding solutions in an online group setting.

However, the authors will make certain changes to the blended teaching that was provided. In future, the blended teaching material will be improved, where podcasts made by the lecturers will replace YouTube videos. To answer any questions the students have on the online content, an additional online discussion session will be introduced, so that students do not have to wait for face-to-face interactions for their questions to be answered. During the hospital visit students will not take a video of their patient management. It was their first time treating patients, and they were not comfortable with their peers seeing these videos. Discussion of their patients and the experiences will be face to face. Future research investigating the knowledge acquisition and carryover of theoretical knowledge into the clinical setting is suggested.

The limitations of our study include the omission of determining the student perceptions of face-to-face teaching. A retrospective analysis looking at comparisons of students’ theoretical and clinical marks for orthopaedic, NMS and CP knowledge areas could not be performed because of incomplete orthopaedic, CP and NMS theoretical and clinical student marks.

## Conclusion

A blended teaching approach significantly improved the students’ theoretical marks in a physiotherapy orthopaedics module, when compared to traditional face-to-face teaching in other areas, but not their clinical performance. The students were supportive of the use of blended teaching when surveyed regarding their experience. With regard to fostering the 21st-century learning skills of critical thinking, communication, collaboration, flexibility, creativity and self-directed learning amongst undergraduate physiotherapy students going into clinical practice, the results from our study appear to be promising. Caution should however be taken to not assume that clinical skills are enhanced with a blended teaching approach.
